# Book Review

**Published:** 1924-07

**Authors:** 


### Reviews of Books.

---

**Text-Book of Tracheo-Bronchoscopy.** By Dr. M. Mann, Senior Physician to Department for Diseases of Ear, Nose and Throat, Municipal Hospital, Dresden-Friedrichstadt. Translated by A. R. Moodie, M.A., M.D., F.R.C.S.E. Pp. 292. Illustrated. London: John Bale, Sons and Danielsson Ltd. 1920. Price £1 11s. 6d.—This is intended to be a practical handbook of the methods and possibilities of Tracheo-Bronchoscopy. It is based on the personal experience of the author, with copious references to the work of others. In this latter citation is made from Austrian, American, French, Italian and Spanish workers; perhaps on account of the Great War, no reference is made to England. Beginning with a very brief account of the anatomy, a considerable space is devoted to the available instruments. A useful table of measurements in the bronchial tree follows, and remarks on the preparation of the patient, etc. The next section of the book is devoted to foreign bodies in the respiratory system, their detection and removal. The author evidently feels this is the most important part of the work, and devotes a third of the whole book to it. A very full account of diseases of the respiratory system from the endoscopic point of view is given, and here the author relies mainly on his own experience; this is a branch of the subject, he says, which has failed to receive the attention it merits. He deals first with diseases of the bronchi, etc., proper, then with the effect on the bronchi of disease in other parts, in each case giving some startling account of endoscopic treatment of these conditions. The third part of the book is occupied with a series of coloured plates, beautifully produced. In the main post-mortem findings are shown. Description is given of each plate on the page opposite. A complete index ends the book. There is an obvious criticism that this subject is too closely related to Oesophagoscopy to be conveniently separated from it. The author himself seems to feel the difficulty at times, for example when dealing with the effect of œsophageal disease on the trachea. Since, however, a writer on the two subjects combined is apt to devote the bulk of the space to the œsophagus, there is certainly room for this work, even though it deals with conditions less commonly seen. Especially notable is the insistence on the possibilities of endoscopy through a tracheotomy opening. Dr. Moodie is to be congratulated on the excellent manner in which this work is presented to us.

**Medical Climatology of England and Wales.** By EDGAR HAWKINS, M.A., M.D., D.P.H. Pp. xiii., 302. London: H. K. Lewis & Co. Ltd. 1923. Price 25s. net.—This manual, which shows evidence of minute care in preparation, is one of those books which are indispensable in the library of the modern physician. For many years the pleasantly discursive but less comprehensive volume of Sir James Clark, published somewhere in the forties, on *The Sanative Influence of Climate*, supplied all that was needed in regard to the comparatively few avowed health resorts of England and Wales. The fuller needs of to-day are well met by Dr. Hawkins' manual, in which the meteorological and geographical factors influencing climate are discussed, the general climate of the British Islands considered, and finally some detailed consideration of the various districts, north, south, east and west, with reference to the chief resorts and their climatic peculiarities, succinctly given. Altogether a very useful and well-produced book.

**A Synoptic Chart of Skin Diseases.** By B. BURNETT HAM, M.D., D.P.H. Pp. 8, with two coloured plates. London: H. K. Lewis & Co. 1923. Price 12s. 6d. net.—The author has endeavoured to portray in his coloured plates a characteristic example of an eruption in a characteristic situation. His two plates represent the anterior and posterior aspects of the human body; upon this single outline the various skin lesions are depicted. On the head are patches of alopecia areata, ringworm, tertiary syphilis, impetigo and the like; on the chest seborrhœa, small pox, pityriasis versicolor, etc.; on the abdomen scarlet fever, measles, pityriasis rosea, X-ray burn; and so forth. The author in his preface points out that the lesions may and do occur in other parts of the body than those illustrated in the chart. The accompanying letterpress gives in tabular form a short description of each eruption, its etiology, common sites, irritation or its absence, other conditions from which the eruption must be distinguished, various notes on the behaviour of the disease and a brief suggestion as to treatment. The compilation has been carefully prepared, but we doubt if the plates and letterpress together will serve to act as anything more than a reminder to a practitioner who is well versed in ordinary skin diseases. To the beginner both the plates and the letterpress may convey but little information. On the whole, the portrayal of the lesions in the plates is not to be compared with modern colour photography as an aid to the study of dermatology.

**Two Lectures on Gastric and Duodenal Ulcers.** By Sir Berkeley Moynihan. Pp. 48. Bristol: John Wright & Sons Ltd. 1923. Price 2s. 6d. net.—We are grateful that the author has published in booklet form the two lectures which he delivered before the Hunterian and Harveian Societies last year. Each lecture is a masterpiece of literary art from the pen of an original worker of vast experience. They should be read and studied by physicians and surgeons alike, since they present the latest views and practice. In the treatment of gastric ulcers the author's predilection is in favour of partial gastrectomy where possible; and for duodenal ulcers he makes frequent use of gastro-duodenostomy, after mobilising the duodenum, combined with excision or infolding of the ulcer.

**High Blood Pressure: its Variations and Control.** By J. F. Halls-Dally, M.D., B.C., M.R.C.P. Pp. xii., 155. London: Wm. Heinemann Ltd. 1923. Price 10s. 6d. net. The author of this essay on high blood pressure has set himself the task of writing a concise summary of modern views of the subject. He has also used his critical faculty to good purpose, supporting this view and discarding that with a good deal of sound judgment. He insists in particular on an accurate measurement of the diastolic pressure, and recommends the auscultatory mode of estimating the pressure. One piece of advice that is especially worth taking to heart is that the observer should make more than one estimation of the systolic and diastolic pressures at a sitting, discarding the first and using the later ones, since these are less liable to nervous exaggeration of the systolic figure. It would be easy to pick out small faults both of omission and of commission in the book, but on the whole it is an adequate and helpful summary of a difficult subject.

**Emergency Operations for General Practitioners.** By H. C. Orrin. Pp. 135. London: Baillière, Tindall & Cox. 1923. Price 7s. 6d.—No doubt there is a real field for some small handy text-book in which a doctor who is confronted by a surgical emergency, on sea or land, shall find not only what he ought to do, but also how to do it. We are acquainted with nothing more suitable for his purpose than this useful little volume. It includes all the ailments he is likely to require to treat, and a few besides; the written description of the necessary steps in the operative or other treatment is a model of lucidity, and the illustrations are both numerous and truly illustrative. The book is well printed and produced. If we might offer a few suggestions for future editions, we would like to see a fuller account of the means of treating suppurative conditions of the hand, making use of the work of Kanavel and Wilkie, and insisting on the importance of a general anæsthetic (not gas), and a tourniquet, when incising for pus. We doubt if a purse-string stitch is the best means of closing a perforated gastric ulcer. In Fig. 36 Macewen's triangle is shown so large that the inexperienced operator who trusts to it will infallibly miss the mastoid antrum.

**Common Infections of the Female Urethra and Cervix.** By Frank Kidd, M.A., Ch.B. Camb., and A. Malcolm Simpson, B.A., M.B. Cantab. Pp. x., 191. London: Oxford Medical Publications. 1924. Price 7s. 6d. net.—In this treatise the authors deal with their subject in all its variation as seen by the venereal expert, the gynæcologist and the physician, and each will find advice of value. The book should be a guide to all those in charge of venereal disease clinics for women; the method of examination, the apparatus necessary and the bacteriological control are all excellent and should be followed. As regards the treatment advocated, this is, in the main, simple; fluorine and potassium permanganate in their various strengths are the chemicals generally recommended, and these applied thoroughly under the guidance of "sight not feel" have, in the authors' hands, effected a cure in the majority of cases both gonorrhœal and non-gonorrhœal. The suggestion that treatment could be carried out by the general practitioner is certainly true, but we doubt the ability of the majority to give the necessary time, and perfunctory treatment is useless. The chapter on prophylaxis is good and fair to both parties in controversy on this vexed question, and the difficulty of prophylaxis in women is pointed out. There is a large amount of repetition in the text. The book should be read by students, general practitioners and specialists, especially those in charge of venereal disease clinics, to whom it is invaluable.

**Defects in Cardiac Rhythm in Relation to Cardiac Failure.** By GEO. A. ALLAN, M.B., F.R.F.P.S.G. Pp. 28. Glasgow: A. Stenhouse. 1924. Price 1s.—This excellent little pamphlet dealing with the effect of arrhythmia on the heart function will supply a much-needed want amongst medical students and general practitioners who have little time for reading the bigger manuals. It is set forth in simple language and is most clear and concise. We cannot agree with the statement on page 23 that complete heart block is the only condition capable of producing such a slow rate, for there are cases of vagal slowing which reach the rate of 35 per minute. This little book can be confidently recommended to anyone who wishes to learn some of the newer work in relation to cardiac disease.

**Wheeler's Handbook of Medicine.** By WILLIAM R. JACK, B.Sc., M.D., F.R.F.P.S.G. Pp. xiv., 629. Edinburgh: E. & S. Livingstone. 1924. Price 12s. 6d. net.—This well-known "cram" book has reached a seventh edition, which is a little larger than its predecessors. The author states that every part has undergone careful revision, and hopes that it may be found as nearly as possible up to date.

**The Student's Hand-book of Surgical Operations.** By Sir FREDERICK TREVES, Bart., G.C.V.O., C.B., LL.D., F.R.C.S., Pp. xi., 552. New York: Cassell & Company. 1924. Price 10s. 6d. net.—This useful little book has been brought thoroughly up to date by Mr. Jonathan Hutchinson. It is unfortunate that students should be called upon to show their surgical skill by performing upon the dead operations which they will never be called upon to carry out in actual practice. This compels every author of such a book as this to devote to purely examinational surgery a great deal of space which might be better occupied. In the present edition the description of this class of work has been considerably curtailed in order to allow the inclusion of the more important operative measures commonly practised upon the living subject. Here and there revision might have been rather more drastic, *e.g.* under "Excision of the knee" one reads "the only indication for this operation is tuberculous disease," and "the operation is usually performed in young subjects," statements which may have been true thirty years ago but which are certainly untrue to-day, when excision for tuberculous disease in young subjects is fortunately almost unknown. The writings of the late Sir Frederick Treves are always so clear, concise and well expressed that they have never failed to appeal to his readers, and this book has proved a good friend to many a student up for his "final." No doubt for many years to come this and subsequent editions will continue to prove "a very present help in time of trouble."

**Ear, Nose and Throat Treatment in General Practice.** By GEORGES PORTMANN, M.D. Translated and Edited by R. SCOTT STEVENSON, M.D. Pp. viii., 180. London: William Heinemann. 1924.—This little book, as indicated in the Preface, does not pretend to present more than an outline of the diseases of the ear, nose and throat. Diagnosis hardly receives any place, and the enormous wealth of prescriptions will appear to the English practitioner to be superfluous. The rather free use of cocaine is to be deprecated. Operative treatment is merely indicated, and in some instances seems to be rather delayed. Like most French books, great care is devoted to the technique of treatment, and some valuable hints may be gained from it. The method of intratracheal administration of drugs by injection with a hypodermic syringe through the cricothyroid membrane is a little in advance of the teaching in this country.

**Diseases of the Nose, Throat and Ear.** Edited by A. LOGAN TURNER, with the collaboration of J. S. FRASER, W. T. GARDINER, J. D. LITHGOW, G. EWART MARTIN, and DOUGLAS GUTHRIE. Pp. xxii., 413. Illustrated. Bristol: John Wright & Sons Ltd. 1924. Price 20s. net.—Dedicated to the memory of Dr. W. G. Porter, who fell in the service of his country shortly after the publication of his work on *Diseases of the Throat, Nose and Ear*, this handbook is produced as the joint work of those engaged in the teaching and practice of the speciality in the Edinburgh Medical School. In order to provide for the senior student and the practitioner within the limitations of a single volume of moderate size, it has required great judgment in preserving a balance in dealing with the subject-matter comprised in the thirty-nine chapters, and the rigid exclusion of any redundant or wordy description. The illustrated description of the clinical anatomy is well done, and adds much to the ready understanding of the pathology and treatment of the various regions. The sections devoted to the newer methods of diagnosis, *e.g.* endoscopy covering œsophagoscopy and bronchoscopy, and the physiology of the vestibular apparatus add much to the value of the work. There are no less than twelve plates, many of these in colour, and 222 black and white illustrations, all excellent and none redundant. It is difficult to distinguish in the merit of the work of the different contributors, for throughout the volume the work is of the highest merit. To cover adequately the extensive ground comprised in diseases of the nose, throat and ear within a volume of this size reflects the greatest credit on the Editor and his staff, and we know of no work on the speciality of the same size which affords such a complete clinical picture of all that could be of practical value to the senior student and practitioner. We cannot withhold praise for the printing, illustrations and everything that pertains to the publisher's department, including very good value at the price. It is a worthy memorial of the young and distinguished author on whose earlier work this much expanded volume has been based.

**The Treatment of Fractures in General Practice.** By C. Max Page, D.S.O., M.S. Lond., F.R.C.S., and W. Rowley Bristow, M.B., M.S. Lond., F.R.C.S. Pp. xi., 239. London: Henry Frowde and Hodder & Stoughton. 1924. First edition. Price 12s. 6d. net.—This book fills the large gap between the formidable modern works on fractures and the sketchy treatment of the subject which is given by the average text-book of surgery—and fills it well. Amid the plethora of old and new methods of treatment advocated to-day we sorely need someone to select for us the *best* means of dealing with each of the common types of fracture. This boon the authors of *The Treatment of Fractures in General Practice* have conferred upon us. With admirable confidence they have omitted regaling us with the traditional methods, now superseded, but which die hard in the text-books. In their place they have introduced us to recent advances tested by their own experience. The practical intention of this manual is shown by a whole chapter being devoted to the somewhat neglected uses of the triangular bandage. A few items call for special mention. For instance, the presence on a bone of a point of maximum tenderness as diagnostic of fracture, usually absent from classical symptomatology, is listed. The importance of full skiagraphic investigation, which cannot be overstressed, receives due emphasis. Diagrams help to contrast the important difference in the destructive effects (and prognosis) of the two types of compound fracture, *i.e.* due to direct or indirect violence. The successful treatment of the transverse fracture of the patella by simple suture of the aponeurosis is new: while the description of treatment of the visceral complications of fractured pelvis is refreshingly clear. Two criticisms may be offered. There is need for expansion in the remarks on manipulative surgery. The public will continue to look too much to the bone-setter for the cure of stiff joints if this part of medical education receives such scant attention. Also when common complications of injuries to the elbow are described neural lesions can hardly be excluded.

